# Robotic Surgery in Gynecology

**DOI:** 10.3389/fsurg.2016.00026

**Published:** 2016-05-02

**Authors:** Jean Bouquet de Joliniere, Armando Librino, Jean-Bernard Dubuisson, Fathi Khomsi, Nordine Ben Ali, Anis Fadhlaoui, J. M. Ayoubi, Anis Feki

**Affiliations:** ^1^Department of Gynecologic and Oncologic Surgery, Cantonal Hospital, Fribourg, Switzerland; ^2^Department of Gynecologic and Oncologic Surgery, Foch Hospital, Suresnes, France

**Keywords:** robotic, laparoscopic surgery, gynecology, minimal invasive surgery, ambulatory care

## Abstract

Minimally invasive surgery (MIS) can be considered as the greatest surgical innovation over the past 30 years. It revolutionized surgical practice with well-proven advantages over traditional open surgery: reduced surgical trauma and incision-related complications, such as surgical-site infections, postoperative pain and hernia, reduced hospital stay, and improved cosmetic outcome. Nonetheless, proficiency in MIS can be technically challenging as conventional laparoscopy is associated with several limitations as the two-dimensional (2D) monitor reduction in-depth perception, camera instability, limited range of motion, and steep learning curves. The surgeon has a low force feedback, which allows simple gestures, respect for tissues, and more effective treatment of complications. Since the 1980s, several computer sciences and robotics projects have been set up to overcome the difficulties encountered with conventional laparoscopy, to augment the surgeon’s skills, achieve accuracy and high precision during complex surgery, and facilitate widespread of MIS. Surgical instruments are guided by haptic interfaces that replicate and filter hand movements. Robotically assisted technology offers advantages that include improved three-dimensional stereoscopic vision, wristed instruments that improve dexterity, and tremor canceling software that improves surgical precision.

## Background and History

Robotic applications to surgery started in 1970s as military projects endorsed by the National Aeronautics and Space Administration (NASA) and funded by the Defense Advanced Research Project Administration (DARPA) in order to replace the surgeon’s physical presence and provide care to astronauts or to soldiers in battlefields (1). From middle 1980s to late 1990s, the first generation of robots was used to perform image-guided precision tasks, but these platforms were limited by basic computer interfaces and required a lengthy preoperative planning. The second and current generation of real-time tele-manipulators was set in a master–slave configuration, in which the master unit (the surgeon’s console) controls a separate slave unit formed by robotic arms with multiple degrees of freedom. Two main tele-manipulators were developed and approved by the US Food and Drug Administration (FDA): the Zeus^®^ system (Computer Motion, Goleta, CA, USA) and the da Vinci^®^ Surgical System (Intuitive Surgical, Sunnyvale, CA, USA). In 2003, Intuitive Surgical acquired Computer Motion, which created a situation of corporate monopoly in the surgical robotics market, a situation that has not changed to date. After the merger of Computer Motion and Intuitive Surgical, development of the Zeus^®^ robot was discontinued in favor of the da Vinci^®^ system, which was equipped with a compact platform moving on wheels, three to four robotic operating arms and a stereoscopic immersive camera being able to give a 10-fold magnified view and under control by the surgeon, for a stable and precise navigation. Ergonomics and dexterity are greatly improved by handles reproducing human hand movements by Endowrist^®^ technology. The da Vinci^®^ Surgical System has been upgraded over recent years to include additional features, such as near-infrared technology, and to facilitate setup. The latest generation da Vinci Xi™ system, released in 2014, is less bulky, and its arms are thinner and arranged in a more ergonomic way, enabling multiquadrant procedures without the need to replace the system. However, lack of force feedback is still one of the major technical drawbacks.

## Penetrance of Robotic-Assisted Surgery in Gynecology

At present, more than 3,200 robotic platforms have been installed worldwide (2,223 United States, 549 Europe, and 494 Rest of World). Approximately 570,000 da Vinci^®^ procedures performed in 2014, up to 9% from 2013 were performed. Only 4 years after its clearance for gynecologic applications, 24% of US gynecologist oncologists reported using robotic-assisted surgery, with 66% indicating that they planned to increase their use of the technology in the next year. The 95% of gynecologic oncology fellows have a robotic platform at their institutions, and 95% were trained to use it (2). According to the same study, 74% of fellows were trained to perform robotic-assisted lymph node dissection, and 44% performed radical hysterectomies (3).

## Clinical Considerations in Gynecology

Since FDA approval of da Vinci^®^ surgical system for use in gynecologic surgery in 2005, the growth of robotic procedures has exploded in recent years. Intuitive Surgical reports that surgeons performed 422,000 operations with its system in the United States in 2013, a 12% increase over 2012 volume. Hysterectomy has been the largest and fastest growing procedure for robotic surgery on the da Vinci^®^ system, having grown 36% in 2 years, to 191,000 cases in 2013. Nonetheless, even though robotic-assisted surgery has been shown to be safe and feasible in gynecological surgery procedures, evaluation in randomized controlled trials comparing it with conventional laparoscopy is limited, and there is a lacking of good evidence to show superiority or clear indication for its use (4). Yet a number of non-medical factors – hospital economics and marketing, surgeons’ desires to embrace technology, and even marketing by the maker of the surgical robot itself – seem to be playing a role in subscribers’ choice of robotic surgery.

## Hysterectomy for Benign Disease

Robotic surgery has gained relatively rapid acceptance in benign hysterectomy in recent years (5). A 2013 JAMA study (6) reported that robotic hysterectomy increased almost 1,000% between 2007 and 2010 – from 0.5 to 9.5% of all hysterectomies – while rates of laparoscopic hysterectomy increased much more slowly, from 24.3 to 30.5%. Rates of robotic hysterectomy were higher in hospitals that had the da Vinci^®^ robot, where they accounted for almost a quarter of hysterectomies. The overall complication rates were similar for robotically assisted and laparoscopic hysterectomies.

As for specific indications for robotic-assisted laparoscopic hysterectomy (RLH), potential benefit has been shown over conventional laparoscopy in obese women (7) and in case of large uteri (8–10). Reduced blood loss, decreased postoperative pain, and shorter hospital stay were associated with RLH compared with vaginal hysterectomy, laparoscopic-assisted vaginal hysterectomy, and total laparoscopic hysterectomy. Furthermore, surgeon preference could be considered as another indication in US as many surgeons are not well trained in laparoscopy and vaginal route and may prefer robotics as it is easier to learn.

To date, there are still limited well-designed studies directly comparing outcomes and costs of conventional laparoscopic hysterectomy with RLH.

The largest retrospective study compared 100 patients treated by conventional laparoscopy hysterectomy before robotic adoption with 100 patients with RLH: the mean operative time (skin to skin) 27 min longer in the RLH group (*P* < 0.001) (3, 11). Longer operative time was confirmed by Nezhat et al. (12). No difference in blood loss, length of stay, and postoperative complications were demonstrated.

The first published prospective study with 40 patients undergoing RLH (13) confirmed a longer operative time over conventional laparoscopy. Even if a slight longer hospital stay was observed statistically significant higher costs were found in RLH patients ($2,861 versus $5,410) in the laparoscopy group.

A randomized study by the same investigators analyzed outcomes and costs of RLH versus laparoscopic hysterectomy in 95 patients and found again a longer operative time for RLH (14). Even if a greater improvement in quality of life 6 weeks after RLH was shown over LSC, there was no difference in postoperative analgesic use or return to normal activities between the groups.

Most investigators consider higher costs per procedure secondary to lengthier operative time and disposable equipment. However, comparative trials can be biased by surgical experience, as operative times are usually shorter in procedures, in which surgeons are expert in comparison to procedures they are new to or they are learning.

Besides operative time and costs, some other technical concerns appear to potentially affect outcomes of RLH in a negative way.

First, vaginal cuff dehiscence is a serious complication of laparoscopic hysterectomy, and robotic assistance could be a major risk factor, possibly due to electrosurgical techniques or vaginal closure technique. In a systematic review (15), RLH is associated with a higher incidence of cuff dehiscence versus conventional laparoscopic hysterectomy (1.64 versus 0.64%). Judicious use of electrocautery at the vaginal cuff by blended cutting electrosurgical current rather than coagulation current and the use of a two-layer cuff closure or bidirectional barbed suture are recommended to decrease the risk of vaginal cuff dehiscence. Second, the safety communication released by the US FDA in September 2014 (16) because of the risk of dissemination of undiagnosed uterine sarcoma in patients undergoing hysterectomies or myomectomies with power morcellation may decrease the rate of RLH and conventional laparoscopic hysterectomy in the US and Europe during the next few years, unless diagnostic tests for uterine sarcoma improve or innovative devices are developed and deemed safe and effective for uterine power morcellation.

In summary, to date, evidence suggests no clear indications for RLH over other routes of minimally invasive hysterectomy for benign disease, for which robotics offer few surgical advantages over laparoscopy in the hands of experts in conventional laparoscopy, at an increased cost. Insufficient data exist comparing robotics to conventional laparoscopy in the hands of non-experienced surgeons. Many gynecologic surgeons who are not experienced in conventional laparoscopy are keen to adopt robotic surgery. Despite lacking of clear indications for use of robotics over other routes of minimally invasive hysterectomy (vaginal and conventional laparoscopy), a great number of women could benefit from minimally invasive hysterectomy because the robot is an easy to learn, rapidly adopted, minimally invasive tool (5).

## Myomectomy

Robotic myomectomy was developed and embraced by surgeons based on success laparoscopy had already achieved. The robotic approach has increased in popularity as it is a suture-intensive surgery, and assistance with robotic arms makes suturing simple and easy and allows surgeons with limited or no laparoscopic experience in suturing technique to perform the procedure in a minimally invasive way. However, there are a few limitations of robotic-assisted myomectomy, such as reduced field of vision and inability to apply torque, which makes removal of very large myomas difficult. Some authors recommend hybrid approach (conventional laparoscopy and robotic assistance) in case of myomas larger than 10 cm, beyond the pelvis, to preserve tactile sensation and avoid robotic equipment damage during traction and dissection (17). Lack of haptic feedback can cause breakage in suture material during suturing, but this can be overcome by increasing experience of the surgeon. Robotic approach may help surgeons to extend their boundaries in terms of size and number of myomas treated by minimally invasive techniques. However, superiority over laparoscopy has not been confirmed by data so far. In retrospective studies, robotic assistance seems to offer less blood loss and hospital stay but longer operative time when compared with conventional laparoscopy (18).

## Endometriosis

When performed by conventional laparoscopy, surgery for endometriosis is perhaps the most technically challenging in gynecology. The dense adhesions, the loss of function of adnexal structures, and the poor reproductive outcome put enormous pressure of the surgeon to restore anatomy and function by removal of all endometriotic implants and improve patient’s quality of life (17). Robotic assistance may make this difficult task easier as a detailed and magnified 3D surgical view can greatly improve the quality of the surgical dissection in complex cases. However, published data are still sparse, and there is no solid evidence in literature to prove that. The largest series of robotic-assisted surgery for deep endometriosis has been published by Siesto et al. (19) in a retrospective cohort study showing neither significant intraoperative complications nor conversion to laparotomy. A retrospective study published by Nezhat et al. (20) evaluated 78 patients with endometriosis and found a longer operative time but no significant difference in blood loss, hospital stay, and complications when compared with conventional laparoscopy. In summary, it seems robotic-assisted surgery does appear to be a feasible, safe alternative to achieve a comprehensive surgical treatment in endometriosis. However, outcome superiority to conventional laparoscopy is still to be demonstrated.

## Pelvic Organ Prolapse Repair

Over the past two decades, minimally invasive surgery (MIS) abdominal surgery has increasingly been used to treat pelvic organ prolapse. Besides the several advantages associated with minimal invasiveness, this approach bridged the gap between the benefits of vaginal surgery with the surgical success rates of open abdominal procedures.

The most commonly performed procedure for suspension of the vaginal apex for postoperative vaginal prolapse by robotic-assisted laparoscopy is the sacrocolpopexy (5). Conventional laparoscopic application of this procedure was first reported in 1994 by Nezhat et al. and had not gained widespread adoption due to lengthy learning curve associated with laparoscopic suturing (21). Since FDA approval of the da Vinci^®^ robot for gynecologic surgery in 2005, minimally invasive abdominal surgery for pelvic organ prolapse has become increasingly popular, as robotic-assisted laparoscopic sacrocolpopexy is an option for those surgeons without experience or training in the conventional route. At present, few randomized studies evaluated outcomes of robotic-assisted sacrocolpopexy versus conventional laparoscopy with robotic groups having longer operative times, short-term higher postoperative pain, and higher costs (22, 23). Complications, anatomical outcomes, and quality of life did not differ in general between the groups (23). Repair of pelvic organ prolapse can be performed robotically, and sometimes surgeons can feel suturing and exposure during the procedures less challenging with the assistance of the robot. However, even if robotic surgery may confer many benefits over conventional laparoscopy, these advantages should continue to be weighed against the cost of the technology. To date, as long-term outcomes, there is very little evidence about minimally invasive abdominal procedures for a repair of pelvic organ prolapse, and much more investigations are needed to evaluate subjective and objective outcomes, perioperative and postoperative adverse events, and costs associated with these procedures (5).

## Gynecologic Oncology

Since the da Vinci^®^ surgical system approval in 2005 for use in gynecologic surgery, there has been a growing body of published reports evaluating the utility of robotic-assisted surgery in gynecologic oncology. Despite the well-known drawbacks associated with robotics and the fact that for many years the da Vinci^®^ platform was not intended for simultaneous multiple quadrant surgery (new da Vinci Xi™ actually is), the technology has been widely adopted and has achieved considerable penetrance within the US gynecologic oncology surgeon community (24). According to the Society of Gynecologic Oncology’s robotic task force position statement, robotic-assisted surgery has indeed “markedly changed” the practice patterns in the US gynecologist oncologist community (25). However, there is still no clear evidence whether robotic-assisted surgery is really superior to conventional laparoscopy in gynecologic oncology.

## Robotic-Assisted Surgery in Cervical Cancer

Abdominal radical hysterectomy (ARH) has been the standard of care for patients with early disease (FIGO stage 1A2-2A). Laparoscopic approach of the procedure has been adopted by only a small number of surgeons because of its complexity. By contrast, radical hysterectomy can be made less challenging by robotic platform and can be considered as an ideal surgery for the adoption of the technology.

Multiple studies have evaluated the feasibility, the safety, and the outcomes of robotic-assisted radical hysterectomy (RRH). Comparative trials have demonstrated similar complications rates, improved operative outcomes, such as estimated blood loss and infections in the RRH groups, and variable operative times if compared with ARH and total laparoscopic radical hysterectomy (TLRH) (26–30). Moreover, it seems that the traditional laparoscopic radical hysterectomy experience is not required to use the robotic-assisted approach (26). Overall, intraoperative and postoperative complications rate does not appear to be significantly different between RRH, TLRH, and ARH, and both minimally invasive approaches (RRH and TLRH) could be associated with fewer requirements in blood transfusions and a shorter hospital stay. Data on long-term outcomes in patients undergoing a RRH are sparse. However, equivalent disease-free recurrence rate appear to be equivalent in the RRH groups over after ARH 24 and 48 months (95 and 97%, respectively) (31). Several reports have demonstrated that the number of lymph nodes retrieved is at least equal, if not larger, in RRH compared with traditional approaches (24).

## Robotic-Assisted Surgery in Endometrial Cancer

Endometrial cancer is the most common indication to use the robotic platform in gynecology oncology. MIS has become the accepted standard of care for endometrial cancer surgical staging. However, evidence from randomized clinical trials comparing robotic-assisted approach and conventional laparoscopy is still lacking, and most data are form retrospectives studies. Benefits robotic technology over laparotomy appear evident in a large prospective trial published by Paley (32), where 377 patients undergoing robotic-assisted hysterectomy had much fewer serious complications, such as wound dehiscence, bleeding and urologic injury, and shorter length of stay at hospital when compared with 131 patients who had open surgery for the same indication. In a systematic review, Gaia et al. found less blood loss in robotic group versus conventional laparoscopy (33). Interestingly, operative times for robotic-assisted surgery were similar to laparoscopy (219 versus 209 min, *P* = ns) but significantly longer than laparotomy (*P* < 0.005). The robotic approach can be challenging in case of lymphadenectomy when assessing of lymphatics above the level of the inferior mesenteric artery is required. However, the similar number of lymph nodes retrieved between robotic-assisted surgery and laparotomy in several studies suggests that the concern does not translate into clinical practice. Benefits of robotic platform have been observed in trials evaluating outcomes between surgical approaches in obese patients. When compared with laparotomy, Seamon et al. (34) have demonstrated that robotics had better wound complication rates (2 versus 17%, odds ratio 0.10, CI95% 0.02–0.43, *P* = 0.002) and fewer major complications (11 versus 27%, OR 0.29, CI95% 0.13–0.65, *P* = 0.003) in obese patients with a body mass index (BMI) more than 35. Moreover, in a comparison trial published by Gehrig et al. (35), robotic-assisted surgery was associated with shorter operative time (*P* = 0.0004) and hospital stay (*P* = 0.001), higher lymph node count (*P* = 0.004), and less blood loss (*P* < 0.0001) over conventional laparoscopic approach in obese and morbidly obese patients undergoing endometrial cancer surgical staging.

## Robotic-Assisted Surgery in Ovarian Cancer

The role of robotic surgery in ovarian cancer is not clear, as published data are still very sparse. Upper abdominal surgery is often required in advanced stages, and at present, the da Vinci^®^ S and Si inability to access the pelvis and the upper quadrants simultaneously has been a major limiting factor to the use of the da Vinci^®^ surgical platform in ovarian cancer. A retrospective review by Feuer et al. (36) evaluated surgical and postoperative outcomes in 63 patients undergoing robotic-assisted surgery for ovarian cancer. A longer operative time, less blood loss, and a shorter hospital stay were shown. Complication rates, lymphadenectomy yields, recurrence risk, and survival at 1 year did not differ between robotic-assisted surgery and laparotomy. To date, surgical centers using the robot for ovarian cancer usually perform surgery only in the setting of isolated recurrences or for diagnostic purposes. Nonetheless, capability to perform multi-quadrant complex surgery is a major asset of the new da Vinci Xi™ surgical platform and could contribute to enhance spread of MIS in ovarian cancer patients in the next few years.

## Costs

In an era of shrinking reimbursements and added financial pressures on hospitals, health-care cost has emerged as a major driving force for the adoption of surgical technology (24). The cost of ownership for a robotic surgery system depends heavily on the combined expenditure of the capital, consumables, and service contract. The current generation of robots costs over $1.5 million upfront, which is a considerable investment for most hospitals. Consumables costs can add an additional $1,500–$3,000 to each procedure, while service costs can be more than $140,000 per year. Based on 150 procedures per year over a 5-year period, this translates to a cost of $4,450–$6,000 per patient. In addition to technology costs, training for both surgeons and clinical staff will also be a large item; training costs start at $20,000 per surgeon and can be higher depending on the skill level of the surgeon and the procedure. In addition, operating and recovery room time, hospital stay, time to return to work, and cost of readmission for complications should all be factored in (24, 37).

High utilization will be the key for a program to have positive margins. Very high volume programs typically perform 200–380 procedures per system, per year, although 150–200 procedures per year are normal for active programs. In many institutions, there are requirements put in place for minimum annual robotic case volumes to maintain privileges. If the institution purchases the platform, it must guarantee its use because of initial cash outlay and cost of upkeep. As previously stated, a number of trials have shown that robotic hysterectomies are more costly than laparoscopic hysterectomies. However, it is likely that cost would go down with decreased operative time and that operative time would go down with increased experience. In the present medico-economic climate, comparative cost has come under scrutiny with robotics due to the increased upfront cost but would actually be applicable for any technology which is new (37).

As any emerging technology, costs will come down as a technology matures and competition enters the market. Next-generation systems are expected to be priced more than $600,000, although some specialty robotic systems could be priced as low as $250,000. This reduction in price could allow providers performing 200 procedures per year to see margins of $1.3 million over a 3-year period, which would make this technology very attractive to lower volume providers.

## Learning Curve

Success in adoption of a surgical modality is highly dependent of the learning curve associated with acquisition of the necessary skills to comfortably and efficiently perform it. For the surgeon, robotic surgery overcomes some problems of conventional laparoscopic surgery. Muscular efforts are minimized because improved ergonomics when sitting at a console separate from the patient. Furthermore, combination of improved imaging and instrument control could allow for a faster surgical learning curve compared with conventional laparoscopy, which includes two-dimensional imaging and counterintuitive hand movements (38). However, even if the use of robot-assisted technology is believed to shorten the learning curve of complex minimally invasive procedures, it has not been fully demonstrated yet in literature. The number of cases required for proficiency in robotic-assisted gynecological surgery is not clear. One retrospective study evaluated robotic learning curves based on time for completion of the index gynecologic procedures (39). Investigators reported that times plateaued after 50 cases. Lim et al. assessed the learning curve associated with performing the first 122 cases of robotic-assisted endometrial cancer surgical staging versus the first 122 procedures performed by laparoscopy (40). The authors demonstrated that 24 cases were required in the robotic arm before stabilization of the operative times occurred, whereas double that many were needed when procedures were performed by laparoscopy. Similarly, a second study showed that proficiency in robotic-assisted hysterectomy and lymphadenectomy for endometrial cancer were achieved after 20 cases (41). A more recent publication by Sandadi et al. evaluated the learning curve for completion of a robotic-assisted hysterectomy and showed that 33 cases were required before a fellow can be proficient in performing the procedure (42).

In summary, robotics may permit less experienced laparoscopic surgeons to perform minimally invasive procedures that previously would have required laparotomy. Factors that affect this learning curve include abdominal or laparoscopic experience with procedures being performed, prior laparoscopic skills of the surgeon, and the experience of the robotic surgical team. Training of the surgical team is essential and has been reported to decrease operative time and complication rates (38, 39, 43).

## Credentialing and Training

Currently, the optimal approach for training in robotic surgery remains undefined. Residents in US obstetric and gynecologic programs approved by the Accreditation Council for Graduate Medical Education are becoming trained in new minimally invasive technologies, with some residency programs instituting robotic training (38). The Council on Resident Education in Obstetrics and Gynecology is developing criteria for training in robot-assisted surgery. Intuitive Surgical is supporting the Robotic Training Network in a 50-site residency pilot program in gynecology. Training also is available at the fellowship level. The American Board of Obstetrics and Gynecology recommends that residency and fellowship programs serve an important role by ensuring their graduates maintain a balanced experience and that the introduction of robotic technology does not limit graduates’ competence in performing vaginal, laparoscopic, or abdominal hysterectomies. Surgeons should be skilled at abdominal and laparoscopic approaches for a specific procedure before undertaking robotic approaches (38).

Hands-on training using the new technology is paramount. Animal subjects or human cadavers in a laboratory setting are useful. Most current robotic credentialing protocols require a live porcine lab. However, this training is expensive and time consuming, and for some, an ethical issue. As simulation capabilities continue to improve, simulation centers will be able to assist greatly in initial training. Health-care institutions often require practitioners’ initial cases to be proctored by a surgeon experienced with this technology. In small institutions where an experienced proctor does not exist, other pathways may need to be considered.

In Europe, residency programs in gynecology very rarely include a structured robotic teaching and training, as most European robotic gynecologic surgeons still train “on the job.” Nonetheless, some research centers and university institutions offer a few days training courses to initiate surgeons to robotic technology. Noteworthy, School of Robotic Surgery at University of Nancy, France, offers an intensive course with national validation and a particular stress on simulation and surgical team training. Moreover, collaboration with Florida Hospital Nicholson Center, Celebration, and Florida is currently under way to elaborate a standardized method for credentialing robotic surgery skills.

## Future Directions

Global annual robotic revenues are currently approximately $4 billion and are expected to grow to $18 billion in 2018. To date, robotic surgery market is dominated by Intuitive Surgical monopoly, but more competition is welcomed by health-care providers in an effort to lower costs and improve efficacy and safety (37).

An increasing number of potential competitors are at different stages of development. Some have developed competitive platforms for abdominal surgery based on a da Vinci^®^ similar global architecture. Others are working on miniaturized platforms. What has been revealed concerning the potential of new robotic platforms is just the tip of the iceberg, as the major companies involved remain confidential about the real extent of robotic platforms (1).

In Italy, a new surgical platform (TELELAP ALF-X) has been developed by Italian research (SOFAR SPA) in collaboration with European Union (Joint Research Centre) and tested by gynecologic oncology department at Gemelli University in Rome, where about 150 gynecological procedures (benign conditions and initial stages of gynecological cancers) have been performed with positive results. ALF_X consists of a surgeon console and a multiport robotic arm platform. Noteworthy, an advanced eye tracking system is used to control the endoscopic vision in three dimensions through sensors, which move the 3D high definition camera around the surgical field by following the surgeon’s eye movements. Moreover, the platform includes a haptic feedback system, reusable endoscopic instruments, and has already been certified for use in gynecology, urology, thoracic, and general surgery procedures in Europe, but upfront cost is still high at $1–$1.4 million (1, 37).

AVRA Surgical is a New York-based company currently developing the AVRA Surgical Robotic System (ASRS). The platform is configured to be modular with up to four arms and a wireless link between the surgeon’s console and the patient-side cart. Modularity could make the system more cost-effective and allows adapting to multiple surgical and image-guided percutaneous procedures.

Recently, Google and Johnson & Johnson have announced that they will team up to develop an advanced, robot-assisted surgical platform. The team effort will involve Ethicon, maker of medical devices.

## Laparo-Endoscopic Single-Site Surgery

Laparo-endoscopic single-site surgery (LESS) is a relatively new MIS technique aiming to reduce surgical trauma, as the abdominal cavity is reached through a single abdominal incision. The main difficulty in *LESS* is the lack of surgical triangulation giving no other option but to use a chopstick technique, and causing a continuous internal and external conflict between operating instruments and the optical instrument. Robotics could effectively overcome this problem with the ability to cross the instruments and simultaneously invert the control panel through software manipulation (1).

Intuitive Surgical has a dedicated platform enabling LESS, called da Vinci sp™ single port robot-assisted surgical system. The device delivers a 3D HD camera and three fully articulating instruments through its 25-mm cannula. While the FDA has already cleared the system for urologic procedures, the da Vinci sp™ Surgical System will not be released to the market till it will be totally compatible with the Xi platform.

Several snake-like robots are currently under development. The flexible architecture and multiple degrees of freedom make this concept the most suitable one for LESS.

One of most advanced and interesting projects is from Titan Medical (Toronto, ON, Canada), which is developing SPORT™ (Single Port Orifice Robotic Technology). The platform should be smaller and less bulky than da Vinci^®^ and include a surgeon remote workstation and a robotic arm. The system is issued from IREP technology, which has been licensed to the company by Columbia University and consists of a 25-mm single access port containing two snake-like operating instruments (6-mm diameter) and a 3D-HD camera. The first targets of SPORT are gynecology, general and urology procedures, and it is expected to be commercially available in 2016 in Europe and in 2017 in US at a price below $1 million. So far, the platform has demonstrated a high level of dexterity and controllability in an experimental setting and a porcine model. Moreover, the company recently signed an agreement with Anne Arundel Medical Center’s Innovation Center in Maryland to develop a comprehensive training program for the use of the platform (1, 37).

In Europe, a tele-robotic system [Single-access Transluminal Robotic Assistance for Surgeons (STRAS)] to perform LESS is currently under development in Strasbourg, France and includes a high-resolution camera, an intuitive haptic interface, and a visual tracking system (1).

## Image Guidance

Real-time image guidance to complement robotically assisted procedures, through the concepts of augmented reality, may become the main revolution to increase safety and effectiveness in MIS in the next years. During surgery, medical imaging (CT, MRI, or 4D ultrasonography) can be overlaid on real-time patient images, obtaining augmented reality which provides surgical navigation through a patient’s anatomy. At present, the main challenge in image guidance applied to soft tissue surgery is associated with the presence of patient’s respiratory movement and the deformation of soft tissues during surgical manipulation, as the perfect superimposition of real and computer-generated images is inaccurate when rigid model of mobile structures are used. To overcome that, a 3D image of the zone of interest is acquired in the operating room and can be updated at any time during surgery. Future innovations will likely conceive real-time MRI systems to obtain a real-time refresh of the patient model with or without robotic assistance. It is easy to forecast a special interest of image guidance technologies in gynecologic oncology complex procedures (1).

## Conclusion

The magnified view, improved ergonomics, and dexterity offered by robotic platforms may facilitate the switch from open to MIS. To date, overall superiority of robotic technology over conventional laparoscopy has not been demonstrated. However, selected procedures (gynecologic oncology, endometriosis, and microsurgical infertility procedures) might benefit from robotic assistance. Higher costs of robotic surgery are secondary to the Intuitive Surgical monopoly and are likely to decrease with future competitors’ robotic platforms.

## Author Contributions

All authors listed, have made substantial, direct and intellectual contribution to the work, and approved it for publication.

## Conflict of Interest Statement

The authors declare that the research was conducted in the absence of any commercial or financial relationships that could be construed as a potential conflict of interest.
